# Being a quantitative interviewer: qualitatively exploring interviewers' experiences in a longitudinal cohort study

**DOI:** 10.1186/1471-2288-11-165

**Published:** 2011-12-13

**Authors:** Sarah Derrett, Sarah Colhoun

**Affiliations:** 1 Injury Prevention Research Unit, Department of Preventive and Social Medicine, University of Otago, Dunedin, New Zealand

## Abstract

**Background:**

Many studies of health outcomes rely on data collected by interviewers administering highly-structured (quantitative) questionnaires to participants. Little appears to be known about the experiences of such interviewers. This paper explores interviewer experiences of working on a longitudinal study in New Zealand (the Prospective Outcomes of injury Study - POIS). Interviewers administer highly-structured questionnaires to participants, usually by telephone, and enter data into a secure computer program. The research team had expectations of interviewers including: consistent questionnaire administration, timeliness, proportions of potential participants recruited and an empathetic communication style. This paper presents results of a focus group to qualitatively explore with the team of interviewers their experiences, problems encountered, strategies, support systems used and training.

**Methods:**

A focus group with interviewers involved in the POIS interviews was held; it was audio-recorded and transcribed. The analytical method was thematic, with output intended to be descriptive and interpretive.

**Results:**

Nine interviewers participated in the focus group (average time in interviewer role was 31 months). Key themes were: 1) the positive aspects of the quantitative interviewer role (i.e. relationships and resilience, insights gained, and participants' feedback), 2) difficulties interviewers encountered and solutions identified (i.e. stories lost or incomplete, forgotten appointments, telling the stories, acknowledging distress, stories reflected and debriefing and support), and 3) meeting POIS researcher expectations (i.e. performance standards, time-keeping, dealing exclusively with the participant and maintaining privacy).

**Conclusions:**

Interviewers demonstrated great skill in the way they negotiated research team expectations whilst managing the relationships with participants. Interviewers found it helpful to have a research protocol in place in the event of sensitive situations - this appeared to alleviate the pressure on interviewers to carry the burden of responsibility. Interviewers are employed to scientifically gather quantitative data, yet their effectiveness relies largely on their humanity. We propose that the personal connection generated between the interviewers and participants was important, and enabled successful follow-up rates for the study. The enjoyment of these relationships was crucial to interviewers and helped balance the negative aspects of their role. Our results suggest that experienced quantitative interviewers endeavour, as do many qualitative researchers, to carefully and respectfully negotiate the requirements of the interview within a relationship they form with participants: being sensitive to the needs of participants and respectful of their wishes - and establishing an ethical relationship.

## Background

Many studies of health outcomes rely on interviewers to collect data directly from study participants. The role of interviewers is crucial for several reasons: interviewers can be the first official 'face' of the study to participants and the quality of their interaction with participants may affect how the study is regarded; participants may be willing to participate (or not) on the basis of their initial interaction with interviewers; and, rightly or wrongly, "The *interviewer *[authors' italics], as the agent of the researcher, is seen to carry the lion's share of responsibility for the outcome of the data collection process"(p.4)[1-3].

Researchers have investigated the interviewers' role in quantitative study recruitment; administration of computer-assisted interviews; questionnaire design to minimise bias; and effect of interviewer continuity on the response rate in longitudinal studies[3-6]. Although studies have focused on the role of interviewers administering highly-structured quantitative questionnaires, little appears to have been published reporting the experiences of interviewers themselves[7,8]. Information about the experiences of quantitative interviewers may provide opportunities for research teams to improve job descriptions to applicants for interviewer positions, and indicate strategies for improved training and on-going support for interviewers involved in collecting data for health outcome studies. This paper presents results from a focus group held with a team of interviewers working on a quantitative longitudinal cohort study of predictors of disability outcomes for a cohort of injured New Zealanders.

### The Prospective Outcomes of Injury Study (POIS)

The Prospective Outcomes of Injury Study (POIS) commenced in New Zealand in late 2007[9]. One of the aims of this longitudinal cohort study was to (quantitatively) identify factors leading to disability following injury in New Zealand. Interviewers were appointed to collect data from participants using highly-structured questionnaires. POIS participants were injured people recruited by the research team via the Accident Compensation Corporation (ACC). ACC manages the provision of no-fault compensation insurance for all types of injury in New Zealand. This insurance scheme was introduced to New Zealand in 1974 to make access to support following injury equitable and not dependent on lengthy and expensive litigious processes; people injured in New Zealand (including visitors to New Zealand) cannot sue for damages resulting from injury except for exemplary damages[10]. In return, injured people are able to receive a range of health and social supports and services via ACC, including: up to 80% of their pre-injury income while unable to work because of their injury, health care, rehabilitation services and lump sum payments for permanent loss of function[10].

POIS participants had injuries likely to require more than a single 'treatment' and more than one week off paid employment (if they were in paid employment prior to injury). Types of injury included fractures, sprains, injury to the head, burns and multiple trauma[11]. Questionnaires were administered to participants by one of a team of 12 (on average) interviewers using Computer Administered Telephone Interviewing (CATI) to 89% of the cohort of 2856 participants; 11% elected to take part by postal questionnaire, and 0.5% by face-to-face interview[11].

Interviewers were first appointed to work on POIS in December 2007 following an advertisement on-line on the University of Otago website and in a regional newspaper. The job description described the type of study and called for people who were willing to work as part of a team, undertake telephone interviewing and enter data into an electronic system for approximately 5 to 15 hours per week at flexible times to accommodate the preferences of POIS participants. The advertisement called for people with excellent organisational and interpersonal skills (including the ability to relate to seriously injured adults), who were able to work during evenings and weekends and had experience operating computers. The appointed interviewers came from a range of backgrounds - some were students looking for evening and weekend work to fit with their studies, some were working in other part-time positions and most had a background in working with the public (e.g. in education, health or service industries). All had very good communication skills.

During POIS recruitment (December 2007 to August 2009), interviewers received a list of approximately 50 names and contact details for injured potential participants from the research team each month. Interviewers were asked to contact each potential participant to discuss the study further. If the person agreed then the interviewer obtained formal oral consent, and undertook the first of a series of four interviews - scheduled for 3, 5, 12 and 24 months after injury. Consequently, soon after POIS commenced, interviewers were recruiting new participants to the study, while also scheduling and undertaking follow-up interviews. Indeed, there was a time when interviewers were simultaneously managing three tasks: new recruits, 5-month follow-up and 12-month follow-up interviews.

The interviews asked participants a wide range of questions about pre-injury, the injury event and post-injury factors. Questions addressed potentially sensitive topics such as socio-demographic characteristics, general health, alcohol and illicit substance use, work, income, spirituality, depression, sexual functioning and disability. The first interview took the longest time to complete (60 minutes on average), as it collected information about peoples' lives and characteristics before injury, as well as questions about the injury event, early outcome and current health status. Follow-up interviews contained many of the same questions - but fewer overall, and took an average of 30-40 minutes to complete.

### The POIS interviewer role

Interviewer training took 2-3 days to complete depending on whether training was being undertaken on a small group or individual basis, and involved working through and becoming familiar with the POIS interviewer manual, listening to interviews and undertaking 'mock' interviews with actors using the CATI system. The interviewer manual itself covered: background to the POIS study, the consent process, making initial contact, communication skills, use of interviewer first-names only with POIS participants in order to protect interviewer privacy, administration of the set-response structured questions, managing difficult situations and stress management. To use the CATI system, interviewers logged into a secure website to access the software containing the questionnaires[12]. The questions were asked of participants by telephone and the responses were simultaneously recorded on-line and downloaded to a secure University of Otago website.

In common with other research teams, the POIS researchers had expectations of interviewers[13]. Training covered issues such as the requirement that comprehensive instructions accompanying certain questions be read out in full to each participant, that questions were asked of participants 'as written' and that they did not paraphrase questions to aid interpretation, and that questions were repeated if participants found responding difficult. In addition, there were expectations regarding: timeliness of interview scheduling, proportions of people recruited and followed-up (on average), and communication style. Accompanying these research team expectations interviewers were expected to be empathetic, sensitive and careful with time-management. Interviewers self-managed the scheduling of interviews: they needed to attempt to contact each potential participant a minimum of five times for the first interview and eight times for follow-up interviews. If contact could not be achieved then the potential participant was sent a postal questionnaire by the research team.

When POIS participants were contactable and interviewed, their experiences of injury were often of difficulty post-injury. The issue of interviewers asking POIS participants about potentially unhappy or distressing events in their lives was an ethical concern for researchers and was addressed in the training. Interviewers had support available in the form of follow-up phone calls to the research team to talk through issues. They could contact a member of the research team at any time if they wanted to talk through an interview or how they were feeling. The research team also had protocols in place for interviewers to follow when interacting with participants who were struggling. Firstly, all POIS participants, regardless of overt expression of unhappiness or distress, were encouraged to contact their health professional if the interview stirred up any issues for them. Secondly, after the interview, all participants (again regardless of overt unhappiness or distress) were sent a list of contact details for local health and social support agencies. If interviewers were concerned about a participant who was overtly struggling (and that person was not currently receiving health care to help address this) they were to refer these participants to the research team for follow-up.

The research team was able to monitor the activity of each interviewer's contact and follow up list. Targets were established for the proportion of potential participants each interviewer was expected to recruit each month. The average interview duration was monitored for each interviewer with the intention of minimising respondent burden and keeping within the study budget. Time allowances were made for administration and contacting participants. Interviewers were asked to notify researchers of extraordinarily lengthy interviews and the reason for these. In order to keep individual interviewers informed of their progress in meeting targets, the research team sent out anonymous monthly spreadsheets showing the proportion of participants interviewed and the average interview length for each interviewer. Further time was budgeted for interviewers to attend in-person or telephone conference call interviewer group meetings, which were held on a regular basis to discuss problems experienced and strategies recommended by interviewers and the research team.

This paper presents results of a focus group to qualitatively explore with the team of POIS interviewers their experiences, problems encountered, strategies for addressing these, support systems used and training.

## Methods

Regular POIS interviewer meetings were held (at least monthly in the early phases of the study) to provide interviewers with the opportunity to discuss together, and with research team members, any issues encountered during interviews. As a result, the research team recognised the value of undertaking qualitative research with the interviewers about their experiences. Ethical approval was gained from the University of Otago for the interviewer focus group. The focus group was held in March 2011, which was near the end of the POIS data collection period. This was opportunistic scheduling, as interviewers were going to be gathered together in one location for a seminar about emerging results from the project.

This was a 'naturally occurring' focus group comprising the team of POIS interviewers still involved in data collection the last year of the study. There were 10 such interviewers, however one was unable to attend. The length of time employed on POIS for the nine focus group participants ranged between 12 and 38 months (average 31 months). In the course of more than three year's data collection for POIS (recruitment of POIS participants commenced in December 2007 and final 24-month interviews were on-going in 2011, at the time of the focus group), other interviewers had been involved in the project but had ceased interviewing a year, or more, before the focus group. Five past-interviewers had moved overseas or begun full-time employment precluding their interviewing; five resigned after 4-18 months employment being unable to continue to work the number of flexible hours necessary for interviewing participants; one resigned for reason of frustration with participants failing to remember scheduled interviews after seven months; another person resigned for unstated reason after eight months, and four had been appointed on temporary contracts to respond to the need to have additional interviewer-capacity at the time new recruitment interviews, 5-month and 12-month interviews were happening simultaneously).

The number of focus group participants was slightly larger than the norm for such groups, which potentially could have disturbed the 'focus' of the group. However, the interview team were all familiar with each other and with the facilitator, SD (study principal investigator). Group introductions were not required and it was expected that interactions would be natural. There had also been time for the group to socialise in a relaxed manner the day before the focus group.

SD coordinated the focus group. A list of proposed focus group topics, developed by SD and SC, was circulated to all potential participants beforehand along with a study information sheet, with an invitation to add to the topics before or during the focus group. Topic areas included: managing the twin tasks of asking 'set questions' from the structured questionnaire and developing rapport with participants; engaging with participants so that they may continue to be willing to participate in the follow-up interviews; accommodating the interviewing role into their everyday life; what sustained and/or supported interviewers; the effect of listening to a variety of life-stories, including tragic ones; and aspects of the interviewer role that could benefit from more attention in the training or training manual and how interviewer-support could be improved.

At the beginning of the focus group, SD explained how focus groups tend to function, noting the importance of there being no 'right or wrong' conversations or ideas, and indeed that people are encouraged to raise and discuss a number of differing ideas and opinions, along with guidelines such as endeavouring not to interrupt each other. Participants completed consent forms including whether or not they were willing to be acknowledged by name in this paper.

The focus group was audio-recorded, then later transcribed by SC, and verified by SD. NVivo software was used to record codes and themes during analysis[14]. SD and SC both generated the coding framework by considering each piece of text and developing a provisional coding framework after familiarisation with the transcript achieved by multiple readings. They then independently coded the transcript, resolving by agreement any coding differences, and creating new codes where these were required. The analytical method was thematic, with the output intended to be descriptive and interpretive[15,16]. In the final stages of analysis, attention was concentrated on areas addressed in this paper. Quotes are provided where they are particularly illustrative. Interviewers are identified by a unique identifier, according to the order of quotes provided in this paper (e.g. *Int1 or Int2*), to enable the reader to observe quotes from the same interviewer. All focus group participants were provided with a draft of this paper to verify that information could not be attributed to them personally, and for suggested improvements to the paper.

## Results and Discussion

Nine interviewers and SD participated in the two-hour focus group. The focus group covered the listed topic areas, although not in sequence, as the flow of discussion within the group was intended to be as natural as possible. Interviewers had valuable insights beyond those reported in this paper, such as what attracted them to apply for the position of interviewer with the POIS study and satisfaction with the CATI system for recording data. The results presented below shed light on themes relating to: 1) the positive aspects of the quantitative interviewer role (i.e. relationships and resilience, insights gained, and participants' feedback), 2) difficulties interviewers encountered and solutions identified (i.e. stories lost or incomplete, forgotten appointments, telling the stories, acknowledging distress, stories reflected and debriefing and support), and 3) meeting POIS researcher expectations (i.e. performance standards, time-keeping, dealing exclusively with the participant and maintaining privacy).

### Positive aspects of interviewing in a longitudinal quantitative study

Interviewers shared examples of positive aspects of interviewing on a longitudinal study. Many of these centred on the value of the interviewer-participant relationship as perceived by the interviewers. Value to the interviewer was not only in terms of the positive relationships forged with participants while interviewing, but also in monitoring the trajectory of the participant's injury-related recovery over time. Many interviewers reported that the insights gained into participants' resilience provided inspiration for their own lives.

### Relationships and resilience

For some, one of the pleasures of the interviewing experience was simply in the relationship gained by 'connecting' with the participant on the end of the phone. Rapport could be quickly established. For example, *"They picked up on my accent, often, and they'd ask me where I came from, and I'd ask them where they came from, and we'd have a wee talk [Int1]*." In some cases, a face-to-face interview was requested by the participant and interviewer and participant met in person: *"Actually, some people you connected with on the phone. You just knew you would actually like to meet these people. And this lady was absolutely utterly lovely - she was such a gorgeous lady, and it was really nice, doing that one face-to-face...It was interesting that kind of rapport, with the odd person. That you just knew you would like.*..*[Int2]" "...I think I could be a friend to that person [Int3]."*

Connections were further enhanced by the longitudinal nature of the project, which meant that interviewers returned to the same participant many times. As a consequence of follow-up interviews with the same participants, interviewers had the opportunity to observe the trajectory of peoples' injury recovery (or not) over time. Interviewers viewed their role as one which provided privileged insights:

"When you re-phoned a participant, [who] when last time you had called was having a really rough time, to find that they had recovered to some extent or fully, [it] always felt great - even though you were not responsible in any way for that recovery. Some peoples' optimism and situations made me at times refocus on my own life. There were times when I thought 'My God - how can someone have such a rough time and still be so optimistic and move forward?'...There are still times when I think of these stories and use these people as role models to my own attitude and life and hope to continue to do so [Int2]."

### Insights gained

Throughout the POIS study with its large cohort, interviewers encountered a range of injury types and coping styles. Interviewers appreciated the insights they gained into how people lived their lives after injury and participants' resilience, and reported personally learning from those insights: *"I quite enjoyed gaining knowledge about various injuries, peoples' struggles, their coping mechanisms, the way people spoke about our research and the opportunity to meet and listen to some inspirational people. The stories are amazing, the people were amazing. I was surprised at the amount of private information that some people were willing to share [Int4]."*

Injuries are prevalent in New Zealand, as they are in many countries. New Zealand has a population of just over 4.3 million people with 1.75 million injury events reported to ACC annually[10]. Perhaps not surprisingly, some interviewers were injured (outside work) over the duration of the data collection. Where this happened, they reported being careful not to overtly empathise with participants: *'It was always in my mind - my own injury, but I just had to hold back, you know - concentrate [Int1]'*. Participants' stories of injury were reported to help put their own injuries into perspective. Another interviewer reported that the interviewer role helped provide a distraction from her own injury-related pain.

### Participants' feedback

At the end of the 24-month interview POIS participants were asked to reflect on their involvement in the study. Interviewers reported satisfaction when collecting comments from participants describing how it was good to have the interviewer listen to their story when their friends or family had stopped listening; and often where recovery was incomplete, being able to reflect on how far they (participants) had progressed over the course of the interviews. Some interviewers wondered if their role was particularly effective, from POIS participants' perspectives, because of the 'anonymity' of the interviewers (being only identified by their first-names). Interviewers thought that being an 'anonymous interviewer' on a phone allowed people to more openly discuss their problems *"A bit like the priest confessional thing [Int5]"*.

### Difficulties encountered in the role of interviewer

#### Stories lost or incomplete

One interviewer spoke about the sense of reciprocity she felt when a participant was interviewed on the telephone while standing outside in the rain:

"I had this one [...] woman, she lived in Auckland, she just had the most amazing personality, and I just loved her, I really did. And um, you know she worked in a factory, I mean what a life! You know, she had [pause], but her attitude was amazing - and our last interview - what happened was that she'd had an argument with her flatmate so it seemed, and then she'd had to go out in the rain and do the interview on her cell phone, but it was really weird, because I felt that she - that I had actually become a good friend of hers. And you know, and that's another one that I was quite sad to let go of [Int6]."

Another interviewer felt the loss acutely because her contract ended prior to all follow-up interviews being completed on her list. Although it was known for some time that the number of interviewers would decrease as the number of 24-month follow-up interviews diminished, the loss was still felt acutely. Other interviewers reported the sense of loss from 'incomplete stories' even when they had undertaken their last 24-month follow-up interview. In this situation, incompleteness resulted from not knowing participants' longer-term outcomes beyond the last 24-month interview.

#### Forgotten appointments

Whilst ending the participant relationship could create a sense of loss for some interviewers, a different sense of loss was experienced by all interviewers when participants failed to keep scheduled interviews. The loss of personal time or missed social opportunities was frustrating for most. Interviewers relied strongly on their personal study diaries to keep records of scheduled interviews. Interviewers were aware, from a regular interviewer group meeting, that one interviewer had left the project quite early on because of an ongoing and increasing frustration with POIS participants' forgetfulness. Interviewers expressed a range of approaches to the problem of 'forgotten interviews'. For example, one interviewer said: *"I used to say to people at the end 'Are you the sort of person that remembers things or do you forget them?' And they'd say 'Oh yeah I forget things' and I'd say to them 'Why don't you go and write that down somewhere [laughter] and they would [Int7]"*.

Others noted the busy lives people lead, and the normality associated with forgetting occasional interviews; and also the legitimate reasons people sometimes had for missing an interview (e.g. taking a sick family member to the doctor or a changed work schedule). The resounding theme was the importance for the interviewer of not taking a negative attitude over a forgotten interview into a re-scheduled appointment with the same participant because "*It shuts things down [Int2]"*. When participants were known by interviewers to be unreliable at keeping appointments, interviewers would arrange to email or to give them a reminder phone call the day before the interview.

The challenge of managing unreliable participants differed according to the interviewing workload. When the study commenced, the workload was great. Interviewers had lists of 50-60 people to try and contact each month and then scheduled follow-up interviews on top of this. Interviewers reported the frustration of managing unreliable participants to be easier when the workload was busier because there were always other people on their list they could try contacting if a participant was not there for a scheduled interview. When the workload tapered off towards the end of the study this became less convenient and interviewers could be frustrated by rushing home from a social event to do an interview with a participant who had forgotten the appointment.

#### Telling the stories

Some interviewers expressed frustration with a reported lack of flexibility within the CATI system. At times they wished to record verbatim information from a participant when they were in a section of the questionnaire that did not relate directly to the important issues the participant was raising. Many interviewers kept pen-and-paper on the desk beside their computer, as they were encouraged to do in training, so they could note things down and enter them later in an appropriate part of the interview where there were 'free text' fields. Sometimes the participants' verbatim experiences simply felt too hard to encapsulate: *"There were some interviews that you had with people and it was tragic - and you can't write that down. You can write down certain comments at the end, but with the study you were never ever going to be able to capture the loss in some peoples' lives through the [highly-structured] format we were doing. And I don't know whether there would ever be any way that you could actually do that. Other than just being the interviewer, and having that relationship with the person, I don't think that could ever have come through [Int2]"*.

## Acknowledging distress

Interviewers were appointed on the basis of their effective communication skills. Interviewers reported that their approach to participants' distress was to acknowledge peoples' loss, and to offer opportunities to complete the interview on another day or take a break. Interviewers reported that participants appreciated being offered the opportunity to pause, and having their experiences acknowledged by the interviewers:

"By the time I got to 'Death of a family member' and I did get a positive response, in some cases, and at that point I'd say 'I'm really sorry to hear that'... [Int6]"; "I said that too... [Int9]"; "That's all I would say... [Int6]"; "You have to..." [Int8]; "Acknowledge it, yeah... [Int2]"; "I'd do that, what you said, make a bit of a comment 'I'm sorry to hear that' [Int5]"; "Yes, that was the least I could do [Int6]".

Sometimes a topic raised by POIS participants was a very sensitive one - such as someone's recent diagnosis of a terminal illness or suicidal thoughts. In such cases interviewers noted that it was helpful to have the research protocols in place. For example: *"Someone...had just tried to kill themselves just five minutes before the interview...the processes you had were really great...I rang [research team] and...just followed the procedures in the yellow book [Interviewer Study Manual] [Int7]"*.

However, the interviewers were hearing participants' stories regularly: *"I had a couple of calls where the stories, as I've said were really awful, and I found the people on the other end of the phone were emotional, and I found one time when I actually got emotional as well, and I was actually crying, and I don't think they knew, but I was actually crying. Because it was just so traumatic for this person - and you can't - you try not to let them know, but I'm sure they can actually feel that as well. You try not to be that emotional, but we're human - sometimes - and this wasn't related to anything that had happened in my life, it was just because this was such a horrible thing to have happened to this person [Int2]".*

### Stories reflected

Not infrequently, participants' life tragedies reflected experiences interviewers had in their own lives. For example, interviewers asked participants structured questions about major life events that had occurred over the past year including deaths in the family, relationship breakdowns and major health issues; these are events which interviewers may also have experienced. Interviewer training emphasised the importance for interviewers of scheduling holidays and break-periods from interviewing regardless of their own life stressors. In addition, interviewers were encouraged to take a break from interviewing when tragic or stressful events occurred in their own lives. Eventually, however, interviewers returned to the interviewer role - albeit when their own sense of loss or grief was still close. Some expressed needing to 'feel detached' at times in their responses to participants: *"And I could understand why they were feeling a certain way, and I found myself [thinking] - 'Oh gosh, detach yourself right now, you're interviewing, you're not a friend, because if I'd been a friend I'd probably be crying with that person right then. But it was hard, to have this invisible thing around you sometimes, it was really important - and I'm quite emotionally stable [Int4]"*.

### Debriefing and support

Whilst interviewers were encouraged to debrief following difficult interviews by phoning the research team, at times some found it difficult, or awkward, to do so. At those times the home-based nature of the work - essentially 'working alone' - was potentially isolating:

"...because it brought up a lot of things for me. And that was when it was really hard to be working at home by yourself, because there was no-one to really talk to. And you could ring and talk to someone, but you don't really want - when you're feeling a bit upset - it's just hard to ring and say 'I'm feeling a bit upset' [laughter] [Int7]".

Interviewers reported adopting informal debriefing strategies. They needed to find a way of debriefing which did not compromise confidentiality. It had been an explicit research expectation that privacy for participants be upheld. Informal strategies included popping next door to a neighbour's and just sharing a cup of coffee as a means of distraction or distance from a distressing interview. One interviewer felt it would be helpful in future training to ask interviewers to nominate a friend with whom they could share how they were feeling without revealing any details of the participant or their injury - but rather sharing how they were feeling as interviewers. Interviewers also met or telephoned one-another regularly for informal support.

### Meeting researcher expectations

The research team had a number of explicit expectations that they emphasised throughout interviewer training, and through written performance standards.

### Performance standards

One such expectation was that the first interview would take one hour, or less, on average, and that interviewers would report reasons for lengthy interviews (e.g. very talkative participants or people finding the set-response structured questions difficult). Learning to manage talkative participants within the boundaries of a structured interview was a challenge in itself. Participants sometimes found it difficult to respond within the set format: *"You [give] them four answers and they choose one that isn't there [Int9]"; "People feel that they want to tell you more than just 'Very satisfied' [Int5]"; "People would want to justify their opinions [Int4]." *Interviewers devised methods to remind participants that they were administering a structured questionnaire: *"[We've] got to choose from one of these, so which one do you reckon [Int5]?" *One strategy that interviewers adopted in helping very talkative participants stay on task was to invite a return to the topic later on in the interview, for example: *"That sounds really interesting I don't have the opportunity to note it now, but we can return to it at the end [Int6]*", and in doing so, managed to sustain rapport and goodwill.

The monthly spreadsheet, showing interviewers their recruitment proportion and average interview times compared to the other (anonymous) interviewers, met with mixed responses from interviewers. Some interviewers saw it as a means of monitoring their own performance month by month, others as a challenge to rise to. Some were simply astonished at what other interviewers were achieving, while others found it 'pressurising' and described difficulty matching the expectation for timely interviews alongside the expectation that they also be sensitive to the needs of participants: *"I remember very strongly feeling it would be insulting to pressurise a participant too much. We had to control the speed of the interview, because you know, it couldn't be open-ended, but I thought, they'd done us the courtesy of setting aside that time, so we couldn't really have it all our own way. We had to give them time to reflect on the question - it's the first time they're hearing it, even though we're saying it for the hundredth time" [Int9]*.

### Time-keeping

Another explicit expectation was that once interviewers had scheduled a time for an interview with participants it was expected that they did not phone these participants back to change the time. Instead, interviewers were asked to notify the research team if they were unable to keep an appointment at the scheduled time, so that another interviewer could do the interview and the participant was not inconvenienced. Several interviewers said that they did rarely reschedule, and they reported that this was never stated to be a problem for participants.

### Dealing exclusively with the participant

Proxy interviews with people other than the injured person were not permitted. This also extended to recruitment where interviewers were required to obtain any refusals to participate directly from potential POIS participants rather than from their family or other household members. On rare occasions a partner would answer the phone and refuse to let interviewers speak to the potential participant for no declared reason, and interviewers reported feeling uncomfortable about this.

### Maintaining privacy

Interviewers were also expected to maintain participants' privacy at all times. At the first interview participants were asked to provide some 'alternative contact' phone numbers in case they moved house, or were otherwise not available at the same phone number, for follow-up interviews. These alternative contacts would then be phoned by interviewers seeking current contact details for the participants. Maintaining participants' privacy, while phoning their alternative contacts, was challenging at times. A strategy used by several interviewers was to identify themselves as working for the University of Otago. They reported that that often provided sufficient legitimacy to satisfy alternative contacts and protect the privacy of study participants: *"You could hear it really in peoples' voices actually - I noticed that there was that sense that you were official. You stood for something quite important really. That was obvious [Int3]"*. O'Brien et al wondered if qualitative research with interviewers may find that stating the affiliation of a respected sponsor (institution) could be sufficient to help recruit participants to surveys[3]. Our results suggest this is indeed the case.

## Conclusions

This paper provides some first insights into the crucial role of the interviewer in a quantitative study. All interviewers reported feeling satisfied with their decision to work on this study. Positive aspects of the interviewer role included opportunities to form relationships with POIS participants within the constraints of the research environment and appreciating the private insights into peoples' resilience - even in the face of poor outcomes. Interviewers discussed strategies they used to help sustain the relationship with study participants such as sharing (anonymous) aspects of their own lives (such as a recognised regional accent) with participants so the interview felt less 'one-sided'. The down-side to feeling connected to participants was the sense of loss when these relationships ended with the final interview or the end of the interviewer's employment contract.

Frustrations encountered by interviewers included the inability of the CATI software to be responsive to the interviewer's wish to record participants stories verbatim, and frustrations when participants failed to remember scheduled interviews. Although this latter frustration proved insurmountable for one interviewer who resigned from the study after eight months, most interviewers reported strategies they used to remind participants of scheduled interviews and acknowledged the generosity of participants taking part when their own lives are busy and/or difficult. Although it did not alleviate the frustration, the research team did discuss the likelihood of participants failing to keep scheduled interview appointments at the time of interviewer recruitment and would recommend this approach to other research groups operating in similar contexts to ours.

Interviewers were mixed in their response to some of the research team expectations - particularly in regard to the use of performance standards. Despite these reservations, the authors do recommend the use of performance standards to help monitor interviewer progress within the constraints of a study budget. Monitoring was also important because once POIS participants consented to participate in the study, the interviews needed to proceed as described in the Study Information Sheet. The monitoring identified changes in performance - and this let the research team work with interviewers to suggest solutions (e.g. time for an interview 'holiday' or transferring some interviews to another interviewer to reduce their interview 'list' for a time). It also allowed the research team to more-easily support and acknowledge the hard work being undertaken by the team who each completed hundreds of interviews over the course of their employment on the longitudinal study.

Interviewers appreciated the protocols established by the research team for interviewer support and dealing with particularly distressed participants. In terms of managing the tragedy of traumatic stories, some developed skills in 'staying detached' from the trauma to minimise the emotional burden of the interview. While other interviewers availed themselves of the opportunities to discuss their own stressful situations with the research team, it sometimes felt that making extraordinary contact with the research team seemed to make 'more' of certain situations, than interviewers felt warranted. Interviewers recommended other strategies that could be useful in future studies such as using informal supports, and perhaps identifying a friend they could talk to about their feelings without revealing any details of the study participants' situation. The overarching finding arising from the interactions between the interviewer focus group was the sense of interviewer respect for study participants within the interview.

While this study provides some first insights from quantitative interviewers about their role, it is not without its limitations. For example, we cannot know, from this focus group, if the apparent importance to interviewers of the sense of interview-relationship with participants was reciprocated or valued by participants. Future planned analysis of the data collected from POIS participants in the 24-month quantitative interview, about the reasons for taking part in POIS, is expected to provide information in this regard. Nor can we know whether the satisfaction expressed by interviewers in observing either the recovery trajectory (where recovery was experienced by injured POIS participants), or, the resilience in the face of non-recovery, would have been as important to interviewers if the POIS project had involved a single cross-sectional interview instead of a series of follow-up interviews. We also cannot know whether it would be similar for quantitative researchers working in other health or disability fields.

The focus group was the method of choice for our study as the team of interviewers were familiar with meeting together to confidentially discuss problems or difficult/unusual situations encountered in their interviewer role. The focus group method also allowed discussion to be generated within and between the nine interviewers - rather than being overly-directed by the facilitator, as may have happened in individual interviews. However, despite the regular team group meetings including both authors, it is possible that SD's involvement as the focus group facilitator may have limited discussion critical of the research team or study process. Future studies may consider using alternative designs such as focus groups or individual interviews conducted by someone independent of the research project. However, it is worth noting that, despite this possible limitation, interviewers in our study did feel free to report negative experiences with the research process.

It is also noteworthy that participants in the focus group were experienced interviewers with an average of 31 months interviewing experience. Five interviewers had resigned after 4-18 months interviewing because they could not sustain the required number of part-time hours to ensure that POIS participants were being interviewed as scheduled, despite the research team's efforts to support them through interview 'breaks' and shorter interviewing 'lists' of participants. The role of quantitative interviewer, on a study such as POIS, requiring people to work flexible part-time hours and undertake independent-scheduling of interviews with participants was not suited to everyone, or at least, not suited to everyone into the longer-term.

To a certain extent, specific training and support strategies could only go some way to address the difficulties experienced as an interviewer. Far more important were the positive relationships interviewers formed with participants in the context of the interview. Indeed, interviewers reported that these relationships and information about recovery trajectories were rewards in themselves. We propose that the personal connection generated between the interviewers and participants was important, and enabled successful follow-up rates for the study. The enjoyment of these relationships was crucial to interviewers and helped balance the negative aspects of their role. Benefits to interviewers were on-going - inspiration from participants reportedly extending for some interviewers beyond the life of the study itself.

Interviewers on this project demonstrated great skill in the way they negotiated research expectations whilst managing the interview relationships. They successfully managed the tension of meeting scientific expectations while engaging in a human way with participants. Interviewers are employed to scientifically gather quantitative data, yet their effectiveness relies largely on their humanity. In training and supporting interviewers, quantitative health researchers can heed this humanity; interviewers are not robots - and nor would it be desirable if they interviewed in an overly static manner. Our research supports calls made from studies with qualitative interviewers about the importance of considering the emotional impact of interviewing on interviewers[17]. Insights provided by the interviewers in the focus group emphasise this actuality, but also indicate strategies, such as lay-debriefing, that could be recommended to future interviewers to help address this burden.

It has been stated that "[Quantitative] Interviewers are trained in the service of the ideal" (p.290)[18]; an ideal whereby researchers envisage interviewers administering questionnaires within a context devoid of the vagaries of the participant. The results of our focus group support an alternative model of the highly-structured quantitative interview. Our results suggest that experienced quantitative interviewers endeavour, as do many qualitative researchers, to carefully and respectfully negotiate the requirements of the interview within a relationship they form with participants; being sensitive to the needs of participants and respectful of their wishes - and establishing an ethical relationship[19,20].

## Competing interests

The authors declare that they have no competing interests.

## Authors' contributions

SD was responsible for the study conception, design, application for ethical approval, data collection, analysis and drafting the manuscript. SC contributed to the list of key topics to be addressed in the focus group, transcribed the interview, co-coded the transcript, helped identify themes during analysis and made critical revisions to the manuscript. Both authors have read and approved the final manuscript.

## Pre-publication history

The pre-publication history for this paper can be accessed here:

http://www.biomedcentral.com/1471-2288/11/165/prepub
